# Gut microbiota and intestinal permeability in rheumatoid arthritis: pathogenic mechanisms

**DOI:** 10.3389/fimmu.2026.1887782

**Published:** 2026-07-15

**Authors:** Jan Bilski, Agata I. Schramm-Luc, Marian Szczepanik, Piotr Pierzchalski, Agnieszka Krawczyk, Kevin Luc

**Affiliations:** 1 Department of Biomechanics and Kinesiology, Chair of Biomedical Sciences, Faculty of Health Sciences, Institute of Physiotherapy, Jagiellonian University Medical College, Krakow, Poland; 2 Department of Internal and Agricultural Medicine, Faculty of Medicine, Jagiellonian University Medical College, Krakow, Poland; 3 Chair of Biomedical Sciences, Institute of Physiotherapy, Faculty of Health Sciences, Jagiellonian University Medical College, Krakow, Poland; 4 Department of Medical Physiology, Chair of Biomedical Sciences, Faculty of Health Sciences, Institute of Physiotherapy, Jagiellonian University Medical College, Krakow, Poland

**Keywords:** dysbiosis, gut micribiota, gut-joint axis, intestinal permeability, mucosal immunity, rheumatoid arthritis, T reg cell, Th 17 cells

## Abstract

Rheumatoid arthritis is a chronic systemic autoimmune disease in which the earliest breaks in immune tolerance may arise at mucosal surfaces before the clinical onset of synovitis. Among these sites, the gut has emerged as a particularly compelling candidate because it integrates microbial, epithelial, metabolic, and immune pathways with the potential to shape systemic inflammation. In this review, we examine the biological foundations of the gut–joint axis in rheumatoid arthritis, focusing on intestinal barrier structure, microbiome alterations, mucosal immune crosstalk, and mechanisms of barrier dysfunction. Current human evidence links rheumatoid arthritis to heterogeneous shifts in gut microbial composition, depletion of beneficial metabolite-producing commensals, altered immune–metabolic signaling, and biomarker patterns consistent with epithelial injury and microbial-product translocation. At the same time, available data do not support the existence of a single, universal microbial or permeability signature that defines the disease across populations. Recent longitudinal studies further challenge the concept of stable, long-standing dysbiosis and instead suggest a late, transient phase of ecological instability arising close to symptom onset. Experimental models provide stronger mechanistic support, showing that dysbiotic microbial communities, impaired barrier integrity, and strain-specific host–microbe interactions can promote T helper 17-skewed immunity and aggravate arthritis. Collectively, these findings support a context-dependent contribution of the gut to rheumatoid arthritis pathogenesis while underscoring the need for longitudinal, strain-resolved, and multi-omic human studies to clarify causality, refine disease models, and identify clinically meaningful windows for intervention.

## 1 Introduction

Rheumatoid arthritis (RA) is a chronic systemic autoimmune disease characterized by synovial inflammation and progressive joint destruction which affects approximately 0.25–1% of adults worldwide, and is more common in women. Although its precise etiology remains unclear, RA is widely considered to arise from a complex interplay between genetic susceptibility and environmental factors (1–3).

In this review we focus on the biological basis of the gut-joint axis in RA: gut barrier structure, microbiome changes, mucosal immune interactions, and mechanisms of barrier dysfunction. Human evidence is prioritized, with preclinical data used to support the mechanistic interpretation. The subsequent section will explore translational aspects, including dietary, microbiological, and lifestyle interventions aimed at sustaining intestinal homeostasis.

Increasing evidence supports the concept that RA may originate at mucosal surfaces, where environmental exposures interact with the immune system to initiate loss of tolerance. In susceptible individuals, this process may precede clinical arthritis and is reflected by the appearance of anti-CCP antibodies and rheumatoid factor (RF) (2–5). While oral microbiota have been implicated in RA pathogenesis through pathobionts such as *Porphyromonas gingivalis (P. gingivalis)* and *Aggregatibacter actinomycetemcomitans (A. actinomycetemcomitans)* (6, 7), the gut microbiota has attracted increasing attention due to its broader immunomodulatory capacity and systemic metabolic influence (8, 9).

Diet, medications (e.g., proton pump inhibitors and antibiotics), smoking, and other environmental factors can alter the composition of the gut microbiota, impair the integrity of the epithelial barrier, and lead to increased intestinal permeability. In susceptible individuals, this may increase exposure to antigens and promote systemic activation of the immune system (3, 10–16).

**Figure 1:**
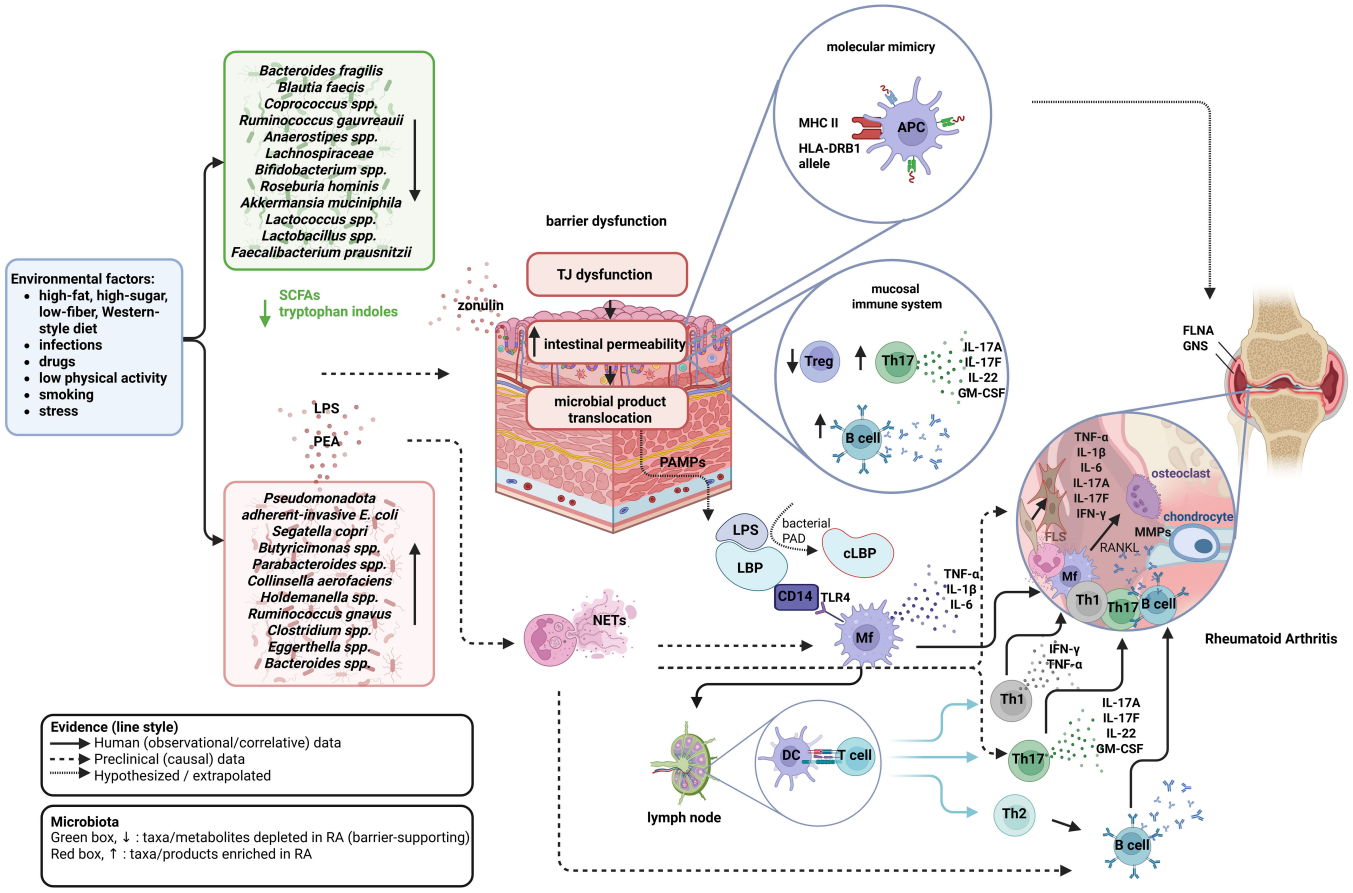
Hypothetical mechanisms linking gut dysbiosis, intestinal barrier dysfunction, and rheumatoid arthritis. Environmental exposures, including a Western-style low-fiber diet, infections, medications, smoking, stress, and low physical activity, may alter the intestinal microbiota and reduce the abundance or activity of short-chain fatty acid-producing, barrier-supporting taxa. Loss of microbial metabolites such as SCFAs may impair tight-junction integrity, promote zonulin signaling, and increase intestinal permeability. Barrier dysfunction may allow translocation of microbial products, such as LPS and other PAMPs, which can activate innate immune pathways through LBP/CD14/TLR4 signaling and induce the production of pro-inflammatory cytokines, including TNF-α, IL-1β, and IL-6. In parallel, dysbiosis may shape mucosal immune responses by promoting Th17-skewed immunity, reducing Treg activity, activating B cells, and supporting antigen presentation by dendritic cells and other APCs. Selected microbial antigens may also contribute to loss of tolerance through molecular mimicry in genetically susceptible individuals carrying rheumatoid arthritis-associated HLA-DRB1 alleles. These converging pathways may amplify systemic inflammation and contribute to synovial immune activation and joint pathology. The model summarizes putative mechanisms supported by variable human associations and stronger preclinical evidence and should not be interpreted as a universal or linear pathway in all individuals with rheumatoid arthritis. APC, antigen-presenting cell; FLNA, filamin A; GM-CSF, granulocyte–macrophage colony-stimulating factor; GNS, N-acetylglucosamine-6-sulfatase; IFN-γ, interferon gamma; IL, interleukin; LBP, lipopolysaccharide-binding protein; LPS, lipopolysaccharide; MHC II, major histocompatibility complex class II; PAMPs, pathogen-associated molecular patterns; SCFAs, short-chain fatty acids; Th, T helper; TJ, tight junction; TLR4, Toll-like receptor 4; TNF-α, tumor necrosis factor alpha; Treg, regulatory T. Created with BioRender.com.

Human studies have reported various gut microbiome alterations in RA, including enrichment of taxa, including *Prevotella* spp. and *Collinsella*, and depletion of butyrate-producing organisms, such as *Faecalibacterium prausnitzii (F. prausnitzii)*; however, no single taxon consistently characterizes the disease across populations, with findings differing by geography, treatment status, and analytical methods (3, 13, 17–21). In medication-naïve early RA, α-diversity, reflecting within-sample microbial richness and evenness, remains unchanged, and β-diversity differences are modest. However, a recent systematic review and meta-analysis of 18 studies reported reduced gut microbial α-diversity in RA overall, with larger effects in treatment-naïve patients, underscoring disease-stage and cohort heterogeneity (22). Metabolomic analyses suggest alterations in microbial tryptophan-derived ligands, including those acting on the aryl hydrocarbon receptor (AhR) (23). Evidence from preclinical RA populations is limited; for example, cohort studies such as SCREEN-RA have failed to demonstrate significant differences in the microbiome before disease onset, highlighting the limitations of cross-sectional and early-stage analyses (24). Longitudinal data suggest that gut microbial changes may represent a late preclinical event, emerging near symptom onset rather than persisting for years (25). These interconnected microbial, epithelial, innate immune, adaptive immune, and joint-directed mechanisms are summarized schematically in **Figure 1**. This model of transient ecological instability helps reconcile conflicting findings and explain why cross-sectional studies often fail to detect consistent signals. Within this framework, *Segatella copri* (formerly *Prevotella copri*) has emerged as a recurrent but dynamic feature of early RA, with its abundance fluctuating rather than remaining elevated (26, 27).

Current human data suggest that certain subsets of patients with RA show microbiome alterations and characteristics of increased intestinal permeability assessed by barrier-related biomarkers, along with immune dysregulation such as Th17 skewing, molecular mimicry, and modified cytokine networks (18, 23, 28–30). However, findings from early-stage and prospective studies have not established clear temporal relationships, leaving the directionality and clinical significance of these observations unresolved (18, 23, 24, 28). Representative human bacteriome profiling studies and preclinical studies of gut microbiota in rheumatoid arthritis are summarized in **Tables 1**, **2**, respectively.

**Table 1:** Representative human bacteriome profiling studies in RA (16S/shotgun).

First author & year	Cohort, sample size & methodology	Key findings & limitations
Scher JU 2013 (89)	New−onset RA vs HC (44 NORA; 28 HC; plus 26 chronic RA & 16 PsA); 16S rRNA V1–V2; shotgun on subset	No α−diversity drop; Segatella copri ↑; Bacteroides ↓; cross−sectional; subset shotgun; single geography
Zhang X 2015 (19)	Early & established RA (multi−site in China); ~66 RA vs 66 HC in one cohort (counts vary); shotgun metagenomics (gut + oral)	Diversity not emphasized; Lactobacillus salivarius ↑ in active RA; Haemophilus ↓; cross−sectional; treatment effects; multi−site sampling
Chen J 2016 (83)	Established RA (USA); 40 RA vs 32 non−RA (15 FDR + 17 HC); 16S rRNA V3–V5	Reduced diversity; Collinsella & Eggerthella ↑; Faecalibacterium ↓; cross−sectional; medication effects
Alpízar−Rodríguez D 2019 (106)	At−risk first−degree relatives; 83 preclinical vs 50 FDR controls; 16S rRNA	No consistent α−diversity signal; Prevotellaceae/Prevotella ↑; cross−sectional; stage heterogeneity
Kishikawa T 2020 (196)	Established RA (Japan); 82 RA vs 42 HC; shotgun MWAS	No significant α/β differences; multiple Prevotella spp. ↑ (e.g., P. denticola); cross−sectional; population−specific signals
Chen Y 2021 (197)	RA vs HC; cross-sectional; stool; 16S rRNA gene sequencing + metabolomics (biomarker discovery)	RA-associated compositional change with linked metabolite signatures; candidate biomarker panel; single-center, needs external validation.
Yu D 2021 (23)	RA vs HC; cross-sectional; stool; 16S rRNA gene sequencing + metabolomics	Altered α/β-diversity and taxonomic shifts in RA with correlated metabolite changes; cross-sectional; medication and diet not fully controlled.
Koh et al., 2023 (97)	Established RA; 94 RA vs 30 HC; 16S rRNA (V4)	Reduced richness in younger RA (<45); Subdoligranulum & Fusicatenibacter ↑ and predictive of treatment response; composition differs from HC; cross−sectional; treatment heterogeneity
Gilbert BTP 2024 (24)	At−risk FDRs across preclinical stages (SCREEN−RA); 371 participants (226 controls; 50 genetic risk; 49 autoimmunity; 46 symptomatic; 8 new−onset); 16S rRNA + quantitative microbiome profiling (QMP)	No significant groupwise differences in diversity or taxa; Prevotellaceae unchanged; cross−sectional within heterogeneous preclinical stages
Rooney CM2024 (25)	Anti−CCP+ at−risk individuals with arthralgia; ≈124 baseline (30 progressors); 19 followed longitudinally; 16S rRNA with targeted shotgun follow−up	Temporal instability (not baseline α−diversity) precedes RA onset; Prevotellaceae shifts contingent on risk/time; small longitudinal subset
Wu et al., 2024 (198)	New−onset RA; 32 RA vs 17 HC; 16S rDNA + untargeted metabolomics + immunophenotyping	β−diversity differences; Ruminococcus 2 ↑; arachidonic−acid metabolites ↓; reduced regulatory T and follicular Tfr cells; small sample; single location
Dunyach-Remy C. 2025 (107)	Recently diagnosed RA (<12 months) n = 25 vs 25 age-matched HC; two French centers; fecal 16S rRNA + stool metaproteomics; plasma 16S rDNA copy number, LBP, sCD14; I-FABP	Dysbiosis with Escherichia↑ and butyrate-producers↓ (e.g., Faecalibacterium, Coprococcus); plasma 16S rDNA↑ and I-FABP↑ → microbial nucleic-acid signatures+ epithelial injury; LBP/sCD14 not consistently↑; single study, modest N

RA, rheumatoid arthritis; HC, healthy control; FDR, first−degree relative; Ps, psoriatic arthritis; NORA, new−onset rheumatoid arthritis; QMP, quantitative microbiome profiling; MWAS, metagenome−wide association study; α−diversity, alpha diversity; β−diversity, beta diversity; Tfr cells, follicular regulatory T cells; LBP lipopolysaccharide-binding protein; sCD14: soluble CD14; I-FABP: intestinal fatty acid–binding protein; ↑, increased/enriched; ↓, decreased/depleted; NR, not reported. Excludes serology/mechanistic, multikingdom (mycobiome/virome), and intervention studies.

**Table 2:** Preclinical studies of gut microbiota in rheumatoid arthritis (animal/*in−vitro* models).

First author & year	Model/intervention	Key findings & limitations
Wu 2010 (151)	K/BxN arthritis; GF)vs (SFB) colonization; Th17 readouts.	GF housing attenuates arthritis. SFB → Th17 expansion and mucosal IL−17 axis; restores disease under GF attenuation. Limitation: model−specific microbial dependency.
Chen 2016 (83)	Experimental arthritis; exposure/gavage with Collinsella aerofaciens; epithelial barrier and cytokines.	↑ gut permeability; ↑ IL−17A; worsened arthritis. Limitation: single−species inoculation; translation to polymicrobial communities uncertain.
Maeda 2016 (154)	SKG arthritis; colonization with Prevotella copri; DCs activation and Th17 response.	Arthritogenic signal; accelerates onset via DCs activation and IL−17. Limitation: SKG−specific susceptibility; strain variability.
Marietta 2016 (199)	HLA−DQ8 humanized CIA; oral Prevotella histicola; mucosal immune and barrier markers.	↓ incidence/severity; ↑ Treg−associated signals; ↑ tight−junction proteins. Limitation: single genetic background; dose/regimen optimization needed.
Rogier 2017 (150)	IL-1Ra−/− spontaneous arthritis; germ-free vs conventional; targeted antibiotics; TLR4/Th17 readouts.	Germ-free or antibiotics attenuate disease; microbiota necessary for full arthritis expression; TLR4-linked dysbiosis. Limitation: strain/model specificity.
Esvaran 2019 (160)	CIA (DBA/1); early oral Lactobacillus fermentum PC1; joint histology; IL−12, IL−4, IL−10.	↓ paw inflammation and synovial infiltration; ↓ IL−12; ↑ IL−4/IL−10. Timing critical. Limitation: one strain; early−intervention bias.
Bai 2021 (200)	CIA; resistant-starch diet; microbiota & SCFA readouts.	Resistant starch increases propionate and alleviates CIA. Limitation: diet-wide confounding; background-strain specificity.
Chriswell 2022 (91)	Human IgA/IgG-selected Subdoligranulum strain; gnotobiotic transfer/functional assays.	Arthritogenic strain identified from at-risk individuals; links mucosal antibody selection to joint-relevant inflammation. Limitation: defined-community context; translation to polymicrobial settings.
Luo 2023 (147)	CIA; antibiotics → fecal transfer from autoantibody-positive at-risk humans; barrier (ZO-1) and Th17 readouts.	Human-to-mouse transfer increases intestinal permeability (ZO-1 perturbation) and Th17; increases arthritis severity. Limitations: antibiotic pre-conditioning; donor heterogeneity.
Seymour 2023 (126)	CIA; microbial indole/tryptophan axis; indole supplementation; cytokines; IL−23 neutralization.	Indole sufficient to exacerbate CIA (↑ IL−6/TNF-α/IL−1β; Th17). IL−23 blockade mitigates effects. Limitation: metabolite−centric; source taxa unresolved.
Moon 2023 (17)	CIA; oral viable Faecalibacterium prausnitzii for 7 weeks; SCFAs and microbiota profiling.	↓ clinical score and joint damage; ↓ IL−17−producing cells; SCFA shift (↓ lactate/acetate, ↑ butyrate). Limitation: small groups; colonization durability uncertain.
Chen 2024 (124)	CIA; defined commensal Bacteroides fragilis; propionate; FLS pathway analyses.	B. fragilis prevents CIA; propionate mediates protection; mechanism maps to HDAC3–FOXK1–interferon programs in human RA FLS. Limitation: single commensal; regimen generalizability.
Kim 2024 (159)	CIA; oral Peptoniphilus gorbachii; barrier integrity (ZO−1, occludin), zonulin, cytokines.	Restores intestinal homeostasis and tight−junctions; ↓ zonulin and pro−inflammatory cytokines; improved scores. Limitation: mediator/metabolite links incomplete.
Li 2024 (92)	In−vitro RA synovial fibroblasts/chondrocytes; Subdoligranulum variabile−derived exosomes; TSG−6, TNF−α.	↑ TSG−6; ↓ TNF−α; anti−inflammatory signal. Limitation: in−vitro only; no in−vivo RA efficacy yet.
Zhang 2024 (201)	CIA; HFD vs control; gut community and metabolomics.	HFD aggravates arthritis with dysbiosis and metabolome shifts; genera–metabolite correlations. Limitation: diet−wide confounding.
Belvončíková 2025 (155)	DBA/1J and Aire^-^/^-^; weekly FMT after antibiotics from healthy/new−onset/relapsing RA donors; 16S V4.	No groupwise severity difference; genus–severity correlations (↑ Paraprevotella/Allobaculum/Phascolarctobacterium; ↓ Odoribacter). Limitations: small N; single 16S region; no sham−FMT.
Borrego 2025 (161)	Pristane-induced arthritis; early-life (post-birth) microbiota transfer.	Perinatal microbiota transfer durably modulates arthritis susceptibility. Limitation: model specificity; narrow timing window.
Han (2025) (144)	Experimental arthritis; Ruminococcus gnavus expansion; metabolite (PEA) analysis; BTK/NET pathway interrogation	R. gnavus produces phenylethylamine (PEA) → activates BTK-dependent NET formation → exacerbates arthritis. Inhibition of bacterial PEA production attenuates disease. Limitation: preclinical; human causality not established; metabolite-driven mechanism requires validation in RA cohorts.
Maeda 2026 (189)	C57BL/6 mice; colonization with Palleniella intestinalis; OMVs stimulation assays	P. intestinalis induces arthritis in genetically resistant mice; ↑ gut permeability; activation of CD11b^+^CD11c^+^ myeloid cells; IL-6–dependent Th17 differentiation; OMVs stimulate DCs to promote Th17 responses. Limitation: preclinical model; human relevance unknown; single-species system.

RA, rheumatoid arthritis; CIA, collagen−induced arthritis; SKG, zymosan−triggered autoimmune arthritis model with ZAP−70 mutation; GF, germ−free; SFB, segmented filamentous bacteria; HFD, high−fat diet; FMT, fecal microbiota transfer/transplantation; SCFA, short−chain fatty acid(s); DCs, dendritic cell; TLR4, Toll-like receptor 4; TJP, tight−junction proteins; ZO−1, zonula occludens−1; TSG−6, TNF−stimulated gene−6; FLS, fibroblast−like synoviocyte; DQ8, HLA−DQ8 transgenic background; PEA, phenylethylamine; BTK - Bruton’s tyrosine kinase; NET - neutrophil extracellular trap; OMVs - outer membrane vesicles. Arrows: ↑ increase/enrichment; ↓ decrease/depletion; → direction of effect (e.g., induces/drives/mediates).

To integrate these observations, this review uses the concept of gut microbial ecosystem instability as an organizing framework. Rather than assuming a fixed RA-specific dysbiosis, we consider whether transient, stage-dependent disturbances in microbial composition, barrier function, microbial metabolism and mucosal immunity may contribute to the transition from systemic autoimmunity to clinical arthritis (25). This narrative review was based on PubMed/MEDLINE and Scopus searches (last search 11 April 2026), prioritizing human evidence and using preclinical studies to support mechanistic interpretation. Translational implications are addressed in the second part of this series.

## 2 The gut barrier in health and disease

### 2.1 Structural and functional components of the intestinal barrier

The gut barrier is comprised of the mucus layer, the epithelial monolayer with junctional complexes, biochemical and immune effectors, and the gut–vascular barrier. Disruption at any level may increase permeability and alter host–microbe crosstalk (31, 32).

The mucus layer that overlies the apical surface provides the first line of physical defense of the gastrointestinal tract and consists of highly glycosylated mucins that contribute to barrier integrity (33–36). Glycosylation is essential for maintaining mucus structure, and its disruption of this process may impair barrier function and increase epithelial exposure to luminal antigens (33, 34, 37–42). It also contributes to epithelial junctional stability (43–48). However, there is currently no evidence for altered intestinal mucin or junctional glycosylation in RA; therefore, links to RA are at the hypothesis-level, extrapolated from Inflammatory bowel disease (IBD) and barrier-injury models (40–42, 48–52).

Mucus in small intestine is relatively permeable compared with colonic mucus, and allows nutrient absorption and bacterial penetration. The mucus layer and the gut microbiota interact bidirectionally, shaping both the barrier properties and the microbial ecology, and this interaction is essential for maintaining barrier effectiveness and overall gut health (53, 54).

Beneath the mucus layer, a polarized epithelial monolayer, consisting of specialized cells originating from crypt stem cells, provides the second line of defense and coordinate interactions with luminal microbes (55–57).

At the apical surface, tight junctions (TJs) seal the intercellular space, while adherens junctions and desmosomes provide structural support and mechanical stability. These complexes are linked to the actin cytoskeleton, enabling regulated barrier function. Tight junctions are composed of transmembrane proteins, such as claudins and junctional adhesion molecules (JAMs), along with cytoplasmic scaffolding proteins, including zonula occludens (ZO) proteins, which anchor junctions to the cytoskeleton and maintain epithelial polarity (57).

Small molecules (⪅20 kDa) may pass paracellularly, whereas larger antigens and microorganisms are transported across the epithelium primarily via specialized antigen-sampling cells or transcytosis (55, 57, 58). Barrier perturbation can also elicit release of zonulin family peptides (ZFPs) from the epithelium, which has been reported to signal through MyD88-dependent pathways and receptor-mediated TJ modulation (59). Antigen sampled by M cells or transcytosed by enterocytes is handed off to the vascularized lamina propria, an immune compartment rich in macrophages, dendritic cells (DCs), and T and B cells that coordinate downstream responses (58, 60).

Beyond the epithelium, the gut–vascular barrier (GVB) further limits systemic exposure to luminal microbial products. Disruption of this barrier, as seen in inflammatory and metabolic conditions, may allow the translocation of microbial products into the circulation (61–63).

### 2.2 Barrier regulation, repair, and dysfunction

In addition to the physical component, the intestinal barrier depends on biochemical and immunological components including bile acids and antimicrobial peptides (AMPs), which help regulate the composition of the microbiota and limit pathogen overgrowth (37, 64, 65). Organized lymphatic structures beneath the epithelium, such as Peyer’s patches, facilitate antigen sampling and immune activation. Specialized epithelial cells facilitate the transport of antigens to the lamina propria, where DCs process and present antigens to initiate adaptive immune responses (11, 66). In this context, microbial antigens can drive T-cell differentiation toward the Th17 phenotype under the influence of cytokines such as IL-6, IL-23, and TGF-β. The Th17-mediated immune response has been implicated in RA pathogenesis, although the extent to which gut-derived signals directly contribute to systemic Th17 skewing in humans remains unresolved (67, 68).

Secretory IgA (sIgA) is produced by plasma cells in the lamina propria and transported to the intestinal lumen via the polymeric Ig receptor. It plays an important role in mucosal immunity by binding to antigens and microorganisms present in the gastrointestinal lumen and limiting their interaction with the epithelium (11, 58, 69).

### 2.3 Hypothetical mechanisms inferred from IBD and barrier-injury models

The Hippo signaling pathway regulates epithelial homeostasis by controlling cell proliferation, differentiation, and tissue repair through the transcriptional co-activators YAP and TAZ, and in healthy epithelium, intercellular junctions restrain YAP/TAZ activity, maintaining epithelial stability and barrier integrity (70–72). Upon epithelial injury or loss of junctional integrity, this restraint is removed, allowing YAP/TAZ activation and promoting regenerative programs that support wound healing and epithelial renewal. Experimental models have demonstrated that transient YAP activation is essential for effective repair, whereas both insufficient and sustained activation can impair epithelial integrity and lead to defective barrier function (73–79).

Disruption of this regulatory balance may result in incomplete maturation of epithelial junctions and increased intestinal permeability. Recent evidence suggests possible interactions between Hippo signaling and glycosylation-dependent junctional stability, in which impaired cell–cell adhesion may facilitate aberrant YAP/TAZ activation and further compromise barrier integrity. While YAP/TAZ activation has been well documented in RA synovium, there is no direct evidence for dysregulation of Hippo signaling in the intestinal epithelium of RA patients, and its role in RA gut pathology remains hypothetical and is largely inferred from studies on IBD and experimental models of barrier injury (70, 71, 79, 80).

## 3 Gut Microbiota and RA

### 3.1 Human evidence for dysbiosis in RA

#### 3.1.1 Recurrent findings in RA-associated microbiome changes

Human studies have described several alterations of the intestinal microbiome in RA; however, these changes are not identical across cohorts. The findings most often repeated include enrichment of *Collinsella aerofaciens* and *Eggerthella* spp., with a relative reduction in butyrate-producing bacteria, particularly *Faecalibacterium prausnitzii* (17, 81). Increased abundance of *Prevotella copri*, now classified as *Segatella copri*, has also been reported, especially in early RA. This association is less stable than was first assumed. It appears to depend on disease stage, treatment status, population background and, probably, strain-level differences (20, 25, 81, 82). This makes it difficult to regard S. copri as a single RA-defining bacterium.

Several RA cohorts have shown a decrease in bacteria usually regarded as barrier-supporting or anti-inflammatory. These include *Faecalibacterium, Roseburia, Blautia, Fusicatenibacter*, and other organisms involved in short-chain fatty acid (SCFA) production (23, 83–87). This observation is biologically plausible, since butyrate and propionate support epithelial integrity and can influence regulatory immune responses. In a study of monozygotic twins discordant for RA, the affected twins had lower abundance of SCFA-producing taxa, including *Blautia faecis*, and lower fecal concentrations of butyrate and propionate than their healthy co-twins (87). This type of study does not prove causality, but it does reduce the problem of genetic confounding and supports a possible environmental contribution of the gut microbiome.

*Collinsella* has attracted particular attention because it may link microbial composition with barrier and immune changes. In a Mayo Clinic cohort, RA patients had reduced microbial diversity, expansion of rare *Actinomycetota*, including *Collinsella* and *Eggerthella*, and depletion of Faecalibacterium (83). Greater Collinsella abundance was associated with increased gut permeability and with a Th17/IL-17A inflammatory signature. In experimental systems, *C. aerofaciens* reduced expression of epithelial tight junction proteins, induced IL-17A-associated gene expression in intestinal epithelial cells, and aggravated arthritis in HLA-DQ8 humanized mice (83). These findings make *Collinsella* biologically interesting, but they do not demonstrate that it is a primary cause of RA in humans.

Perturbation of both oral and gut microbiomes has also been described in RA. Zhang et al. reported mucosal dysbiosis related to disease activity and to serum markers such as CRP, RF and anti-CCP (19). *Haemophilus* species were depleted across mucosal sites, whereas Lactobacillus salivarius was enriched in active RA and appeared more abundant in patients with greater disease activity (19). Functional metagenomic analyses suggested enrichment of microbial pathways related to anaerobic respiration, menaquinone synthesis, redox reactions and metal transport. The same work also identified microbial genes with sequence similarity to host antigens such as type XI collagen (19). These data suggest that RA-associated dysbiosis may involve microbial function as much as taxonomy.

Some findings remain difficult to interpret. The role of *Lactobacillus* is a good example. Several studies have reported increased Lactobacillus abundance in RA, particularly in early disease, whereas others have found a reduction when compared with healthy controls (84, 88–90). The same problem applies to genera that contain strains with different biological effects. *Subdoligranulum* illustrates this well. *S. didolesgii* has been linked with arthritogenic immune responses, whereas *S. variabile* has shown protective effects in experimental models (91, 92). A genus-level increase or decrease may therefore hide biologically opposite strain-level signals.

Microbial metabolites provide another way of linking dysbiosis with immune regulation. In medication-naïve RA, Yu et al. found altered fecal microbiota, modest β-diversity separation and unchanged α-diversity (23). They also found depletion of several tryptophan-derived metabolites, including kynurenic acid, xanthurenic acid, 3-HAA, 5-HIAA and N-methylserotonin. *Escherichia* and *Klebsiella* were closely related to these metabolic shifts in correlation analyses (23). These findings are compatible with a microbiome-metabolite-immune axis, especially through AhR-dependent pathways, but the direction of the effect remains uncertain.

Molecular mimicry is another possible mechanism by which intestinal microbes may contribute to loss of tolerance. Pianta et al. identified filamin A (FLNA) and N-acetylglucosamine-6-sulfatase (GNS) as RA autoantigens, and the immunodominant epitopes showed sequence homology with peptides from gut *Butyricimonas* (93). *S. copri*-derived Pc-p27 has also been shown to induce T-cell reactivity in a proportion of new-onset RA patients and IgA and/or IgG responses in some RA cohorts (94, 95). Lipopolysaccharide-binding protein (LBP), a host chaperone for LPS, can be citrullinated and targeted by autoantibodies in RA (96). Whether gut-derived LBP is the main source of these autoantibodies, or only a bystander in systemic autoimmunity, remains unknown.

#### 3.1.2 Study heterogeneity and methodological limitations

The interpretation of human microbiome studies in RA is complicated by the considerable variation between cohorts. Geography, diet, smoking, medication exposure, age, disease duration, disease activity, autoantibody status and the way in which samples are collected are all likely to influence the intestinal microbiome. These are not simply small technical differences between studies. Smoking has been shown to alter gut microbial composition, and is also a recognized environmental risk factor for RA, in part through effects on protein citrullination and ACPA formation (16, 84). Medication exposure is also likely to be significant. Antibiotics, DMARDs, proton pump inhibitors, and NSAIDs may alter the microbiome, the intestinal barrier, or both (81, 97, 98). This makes comparison between studies difficult, especially when treatment-naïve, early RA and established RA cohorts are considered together.

There are also important differences in the sequencing methods used. Many studies have relied on 16S rRNA gene sequencing. This has been useful in identifying broad differences in bacterial communities between RA patients and controls, but it often cannot identify bacteria at the species or strain level. This limitation is particularly relevant to RA, where strain-level variation may have biological significance. Nii et al. showed that RA-associated *S. copri* strains carried a conjugative transposon region, CTnPc, which was enriched in strains isolated from RA patients and absent from strains associated with controls (20). A similar problem is seen with *Subdoligranulum*, where strains within the same genus may have different immunological properties (91, 92). These observations suggest that genus-level data alone are unlikely to be sufficient, and that shotgun metagenomics, isolation of strains and functional assays are required to determine whether the organisms described in RA studies have pathogenic relevance.

Conflicting genus-level findings in RA should be interpreted with caution when the taxon in question is not well resolved by amplicon sequencing (99). Lactobacillus illustrates this problem well, since older 16S-based studies often grouped together organisms that are now placed in several closely related genera within the Lactobacillaceae family (99). Short V3-V4 reads also have limited species-level resolution in this group, particularly in mixed microbial communities (100).

A similar problem applies to *Subdoligranulum*, with the additional difficulty that the same genus label may include biologically different lineages (91). This may include provisional arthritogenic isolates as well as strains with apparently protective effects, which could partly explain why the direction of association differs between cohorts (91). For studies in which the biological question concerns disease-associated taxa such as *Lactobacillus* or *Subdoligranulum*, shotgun metagenomics would therefore be more appropriate than genus-level 16S profiling alone (91, 101). Strain-aware analysis, metagenome-assembled genomes and, where possible, isolate recovery are likely to be more informative than amplicon profiles by themselves (91, 99). If deep whole-metagenome sequencing is not feasible, shallow shotgun sequencing may be a reasonable alternative, although this point would need support from an additional methodological reference on shallow shotgun sequencing rather than from the present references alone (102).

The diversity data are also difficult to interpret. Some studies, including systematic reviews and meta-analyses, have reported reduced microbial α-diversity in RA, with the strongest effects seen in treatment-naïve patients (22). Other studies of medication-naïve early RA have found no clear difference in α-diversity and only modest separation in β-diversity (23). This suggests that diversity by itself is unlikely to be a reliable biomarker of RA. It may still be informative, but only when considered together with disease stage, treatment exposure and functional microbial changes.

Most human studies have been cross-sectional, and this remains a major limitation. Such studies can show that the microbiome differs between groups, but they cannot determine whether dysbiosis precedes RA, develops as a result of inflammation, or reflects treatment and lifestyle. This is particularly relevant in established disease, where long-standing inflammation and long-term therapy may both reshape the microbiome. In one larger cohort of established RA, the gut microbiome remained distinct from that of healthy controls despite long-term DMARD exposure. Moreover, younger RA patients (aged <45 years) had lower microbial diversity and altered community structure when compared with older patients (97). Baseline enrichment of *Subdoligranulum* together with *Fusicatenibacter* predicted a better 6-month response to second-line csDMARDs (97). While this observation is potentially clinically useful, it also shows that microbiome signals may reflect disease course and treatment response, rather than the original cause of disease.

Genetic causal inference has so far provided only limited support for conclusions based on individual taxa. In a two-sample Mendelian randomization study using MiBioGen genus-level exposures and a large RA GWAS, Wei et al. found only nominal genus-level associations, none of which remained significant after correction for multiple testing (103). Although the direction of effect was consistent across methods and there was no clear evidence of pleiotropy or weak-instrument bias, these signals should be regarded as hypothesis-generating rather than causal. At present, Mendelian randomization provides only weak support for a primary causal role of any single gut taxon in RA and may be more useful for prioritizing microbial pathways or functions for experimental study.

Most work has focused on bacteria, although this may give an incomplete view of host-microbe interactions in RA. Multi-kingdom profiling has reported changes in the mycobiome and virome, including lower abundance of common commensal yeasts such as *Saccharomyces cerevisiae* and *Candida albicans*, and changes in bacteriophage families (104). Other studies have shown altered fungal community structure and bacteria-fungi correlations (105). These findings require replication, but they raise the possibility that bacteria alone may not account for the full microbial contribution to RA.

The above studies suggest that there is unlikely to be a single RA microbiome. A more likely interpretation is that several partially recurring microbial and metabolic changes are present in some patient groups and at some stages of disease. This variation should not necessarily be dismissed as noise. It may indicate that the gut-joint axis is active only in selected patients, at particular time points, or through microbial functions that are not well captured by broad taxonomic surveys.

#### 3.1.3 Insights from longitudinal and at-risk studies

Studies of individuals at risk of RA are particularly useful, since they may help to separate early mucosal events from changes that arise as a consequence of established inflammation and treatment. In first-degree relatives and other at-risk groups, enrichment of *Prevotella* spp. or *Prevotellaceae* has been reported in some cohorts (106). Alpízar-Rodríguez et al. described *Prevotellaceae* signals in first-degree relatives of RA patients, supporting the possibility that mucosal changes may be present before the development of arthritis (106). However, these findings have not been reproduced in all cohorts.

The SCREEN-RA cohort is especially relevant because it examined different preclinical stages of RA (24). This study included first-degree relatives stratified by genetic risk, autoantibody positivity, arthralgia and new-onset RA. No significant differences were found in gut microbiome composition, diversity, *Prevotellacea*e abundance or fecal calprotectin across most preclinical stages. A weak, non-significant *Prevotellaceae* signal was present only in the subgroup with extreme autoimmunity (24). This data argues against the presence of a stable dysbiosis that is easily detected throughout the preclinical period.

Longitudinal studies suggest a somewhat different possibility. In anti-CCP-positive at-risk individuals, Rooney et al. found that progressors had greater baseline *Prevotellaceae* abundance than non-progressors and developed microbiome instability before the onset of clinical arthritis (25). This instability appeared approximately 10 months before arthritis developed. This observation is interesting because it suggests that microbiome changes may occur relatively late, and may fluctuate, rather than being stable for many years before disease onset. It may also explain why cross-sectional studies have often failed to find consistent microbial signals.

This proposed peri-onset period should be interpreted cautiously. The finding comes from one longitudinal anti-CCP-positive cohort with a limited number of progressors and needs to be confirmed in larger independent studies (25). The difference between SCREEN-RA and Rooney et al. may reflect sampling frequency, genetic background, environmental exposures, autoantibody burden, or the possibility that microbiome instability occurs only in selected high-risk subgroups (24, 25). Stool sampling may also miss changes taking place closer to the intestinal mucosa since stool and mucosal microbiome profiles may differ (58, 59). These issues make it premature to define a fixed preclinical microbial signature.

On this evidence, *Prevotellaceae* and *S. copri* remain relevant but should not be treated as universal markers; they more likely represent one component of a broader, time-dependent disturbance involving microbial composition, strain variation, metabolite production, barrier integrity and mucosal immune activation (20, 24, 25, 81, 82).

Future at-risk studies should use serial sampling rather than single stool samples (24, 25). They should combine shotgun metagenomics, metabolomics, fecal and serum markers of barrier function, autoantibody profiling, and mucosal or systemic immune readouts (20, 24, 25, 58, 59, 68, 107–109). This would help to distinguish local intestinal events from systemic inflammation, and to determine whether there is a short period during which gut-directed intervention might be biologically justified. Until such data are available, human studies support an association between RA and altered gut microbial ecology, but not a simple sequence in which persistent dysbiosis alone drives disease development (24, 25, 81, 103, 110).

Taken together, these studies suggest a possible sequence in which the loss of barrier-protective metabolites, SCFAs and tryptophan-derived AhR ligands, together with pro-inflammatory microbial signals such as phenylethylamine and translocated microbial products favors NET formation, macrophage activation and FLS activation, and through these pathways may contribute to synovitis and bone erosion (111, 112).

The main limitation is that the human evidence remains largely associative. Metabolite-microbiome relationships and circulating NET markers can be linked with disease activity, but they do not establish directionality. The strongest causal evidence still comes from collagen-induced arthritis (CIA) models, gnotobiotic systems and human cell-line experiments. This distinction is important, since it argues for caution in making mechanistic claims, while still supporting further studies in human RA based on defined metabolites and functional immune readouts (111–113).

### 3.2 Microbial metabolites, mucosal immunity and loss of tolerance

#### 3.2.1 Metabolite-dependent regulation of mucosal immunity

The compositional changes described above are likely to be biologically relevant only if they result in altered microbial functions that are recognized by the host. One possible way in which this may occur is through microbial metabolites. Short-chain fatty acids, tryptophan-derived metabolites, bile acid derivatives and polyamines have all been shown to act on epithelial cells, dendritic cells, macrophages and lymphocytes (114–122). These metabolites are unlikely to act as single independent signals. Their effect will be influenced by the inflammatory state of the tissue, the integrity of the epithelial barrier and the type of immune cells present in the intestinal mucosa.

Short-chain fatty acids have generally been considered to support epithelial barrier function and to have immunoregulatory properties. Butyrate is an important energy source for colonocytes, contributes to the maintenance of epithelial integrity and may favor regulatory immune responses through GPCR signaling and inhibition of histone deacetylases (115, 119–123). Propionate has also been shown to regulate inflammatory pathways that may be of relevance to arthritis in experimental systems (124). This provides a possible mechanism by which a reduction in SCFA-producing commensal bacteria could be involved in RA. However, the taxonomic evidence for such a reduction has already been considered in **Section 3.1.1**. In humans, the connection between reduced SCFA production, mucosal immune disturbance and the onset of RA remains indirect.

Tryptophan metabolism is another pathway by which the microbiota may influence mucosal immune responses. Several microbial tryptophan metabolites signal through the aryl hydrocarbon receptor (AhR), which is involved in epithelial homeostasis, mucosal Treg responses and the regulation of IL-22 and Th17-related pathways (23, 117, 125). Reduced production of AhR-active metabolites could therefore reduce mucosal tolerance. However, this pathway is not necessarily protective in all situations. In experimental arthritis, selected tryptophan-derived metabolites have been shown to increase inflammatory cytokines and Th17 responses under specific conditions (126). This suggests that the pathway is biologically relevant, but its role in RA is not straightforward.

Other microbial metabolites may also contribute to immune regulation. Bile acid derivatives can influence Treg and Th17 differentiation through nuclear receptors and related immune pathways (125, 127). Polyamines are involved in epithelial repair and in the maintenance of mucosal integrity (114). Histamine has also been suggested as a possible link between microbial metabolism, cytokine production, osteoclastogenesis, and synovial inflammation in RA (128). These mechanisms are less well established than those involving SCFAs or tryptophan metabolism. They do however support the view that dysbiosis may alter immune regulation through changes in microbial function as well as through changes in bacterial abundance. A change at the genus level does not necessarily predict the metabolic activity of the community, since bacteria within the same taxon may have different enzymatic capacities, while unrelated organisms may produce similar metabolites. Future studies will need to combine microbiome profiling with metabolomics and immune readouts, rather than rely on bacterial abundance alone.

### 3.3 Mucosal immune activation and breach of tolerance

The intestinal microbiota is involved in the normal education of mucosal immunity. Recognition of commensal bacteria through Toll-like receptors and other pattern-recognition pathways helps maintain colonization resistance and calibrates immune responses to luminal antigens (114, 129–137). The microbiota also shapes intraepithelial lymphocytes, group 3 innate lymphoid cells, mucosal-associated invariant T cells and lamina propria Th17 cells (132–139). Under physiological conditions, these responses contribute to host defense. In the presence of dysbiosis or barrier injury, the same pathways may contribute to inflammatory immune activation.

Treg cells are central to mucosal tolerance. Microbial products and metabolites can promote Foxp3 expression, support tolerogenic dendritic cells and increase anti-inflammatory cytokines such as IL-10, IL-35 and TGF-β (125, 132, 137–140). These effects have been shown most clearly in experimental systems. In human RA, the evidence is less direct, although altered Treg or T follicular regulatory cell function has been associated with gut microbial and metabolic changes (103, 104, 131). This supports a possible connection between intestinal dysbiosis and impaired immune regulation, but does not show causality.

Th17 responses represent another important pathway. IL-17-producing cells are required for mucosal defense, especially against extracellular fungi and some bacteria (135, 136). During inflammation, IL-1β and IL-23 can promote pathogenic Th17 differentiation, with plasticity towards Th17/Th1-like phenotypes (136). In RA, IL-17A is present in synovial tissue and can activate synoviocytes; however, randomized trials of IL-17 blockade have shown limited or inconsistent benefit in RA, and current EULAR and ACR guidelines do not recommend IL-17 inhibitors for RA. This contrasts with the established efficacy of IL-17 inhibitors in psoriatic arthritis and axial spondyloarthritis (96, 125, 132, 134–142). Although RA and SpA may share some mucosal immune features, they are therefore unlikely to be driven by the same dominant effector cytokine pathways.

Loss of tolerance may also involve antigenic overlap between microbial and host structures. This has been suggested for several bacterial antigens and RA-related autoantigens, including mechanisms discussed above in relation to *S. copri*, Pc-p27, filamin A, GNS and citrullinated LBP (93–95, 114, 143). For the present discussion, the important point is not the identity of each organism, but the broader possibility that mucosal antigen recognition may contribute to systemic autoreactivity. Current human data support this as a plausible mechanism. They do not show that molecular mimicry is a common initiating event in RA.

IgA responses are also consistent with mucosal immune involvement. Microbe-specific IgA or IgG responses in RA and in at-risk individuals suggest that the immune system has been exposed to selected microbial antigens at mucosal sites (94, 95). The significance of these responses remains difficult to define. They may be pathogenic, compensatory or secondary to increased antigen exposure after barrier disturbance. Their timing in relation to the development of clinical arthritis is also still uncertain.

### 3.4 Gut-derived microbial signals and synovial effector pathways

#### 3.4.1 Neutrophil extracellular traps as a point of convergence

Neutrophil extracellular traps (NETs) are increasingly implicated in the link between innate immune activation and the citrullination-autoantibody cycle that is central to RA. Neutrophil extracellular traps contain citrullinated proteins and therefore provide a source of autoantigens that may contribute to the generation of anti-citrullinated protein antibodies (ACPA). They may also act as damage-associated molecular patterns, activating TLR9 and RAGE on macrophages, dendritic cells and fibroblast-like synoviocytes (FLS), with subsequent production of IL-1β, IL-6, TNF-α and type I interferon. NET-derived signals have also been reported to increase IL-17 production by Th17 and γδ T cells, suggesting the existence of a self-sustaining Th17-NET loop, and to promote RANKL-dependent osteoclastogenesis and bone erosion (112).

Rheumatoid arthritis fibroblast-like synoviocytes (RA-FLS) have been shown to internalize NETs through a RAGE-TLR9-dependent pathway. After uptake, they can process NET-derived citrullinated peptides for presentation and respond with increased proliferation and release of pro-inflammatory mediators (112). These findings bring together human observations and experimental data, but the evidence in patients remains mainly associative. For example, circulating NET markers correlate with disease activity and ACPA status, but this does not by itself demonstrate causation. A more direct gut-to-NET mechanism has been proposed from the observation that phenylethylamine derived from *Ruminococcus gnavus* activates a Bruton tyrosine kinase-dependent pathway of NET formation (144). This remains a preclinical, metabolite-driven mechanism, and its relevance to human RA has not yet been established.

### 3.5 Microbial metabolites and macrophage activation

Microbial metabolites may influence macrophage activation in different directions. Short-chain fatty acids, particularly butyrate, and tryptophan-derived aryl hydrocarbon receptor (AhR) ligands are generally associated with reduced NLRP3 inflammasome activation and a more regulatory macrophage phenotype. By contrast, translocated lipopolysaccharide and NET-derived damage-associated molecules favor a pro-inflammatory response, with release of IL-1β, IL-6 and TNF-α (112).

Direct evidence that a defined gut-derived metabolite reprogrammes synovial macrophages in RA is still limited and comes mostly from preclinical systems. For this reason, this pathway should be presented as biologically plausible rather than as an established mechanism in patients.

#### 3.5.1 Microbial tryptophan metabolites and fibroblast-like synoviocytes

The clearest recent evidence linking a gut-derived metabolite with FLS biology comes from studies of microbial tryptophan indoles. In CIA mice and in the human MH7A synoviocyte line, indole-3-propionic acid and indole-3-acetic acid stabilized phosphatase and tensin homolog (**PTEN**) by reducing its ubiquitination (145). This was accompanied by suppression of PI3K-AKT and NF-κB signaling, reduced FLS proliferation, migration and invasion, and increased apoptosis. At the same time, intestinal barrier proteins, including claudin-1 and ZO-1, and AhR signaling were restored (145).

Because these findings combine an animal model with a human FLS line, they go beyond simple correlation. They nevertheless remain preclinical and should not be treated as direct proof of the same mechanism in human RA.

#### 3.5.2 Diet, metabolites and the metabolite-immunity interface

Diet-dependent and microbiome-dependent metabolites provide another example of how intestinal changes may affect the joint indirectly. Fecal biotin has been reported to be increased in patients with RA and to correlate with dysbiotic bacterial taxa. In CIA mice, restriction of biotin reduced arthritis incidence, restored microbial diversity, altered tryptophan metabolism, including 3-indolepropionic acid, and protected against inflammation-related bone loss (146). In this case, the human data are correlative, while the causal argument comes from the animal model.

Taken together, these studies suggest a plausible sequence in which gut-derived metabolites, including phenylethylamine, tryptophan indoles and SCFAs together with translocated microbial products, influence NET formation, macrophage activation and FLS behavior. Through these pathways they may contribute to synovitis and bone erosion.

The main limitation is that the human evidence remains largely associative. Metabolite-microbiome relationships and circulating NET markers can be linked with disease activity, but they do not establish directionality. The strongest causal evidence still comes from CIA models, gnotobiotic systems and human cell-line experiments. This distinction is important, since it argues for caution in making mechanistic claims, while also supporting further studies in human RA that are anchored in defined metabolites and functional immune readouts.

### 3.6 Experimental evidence and strain-specific mechanisms

#### 3.6.1 Microbiota dependence in arthritis models

Experimental arthritis models have provided stronger evidence for a functional role of the gut microbiota than most human studies. This is largely because microbial communities can be removed, transferred or replaced in experimental systems, which is usually not possible in patients. These models do not reproduce the full complexity of human RA but still they have been useful for examining whether intestinal microbes can influence mucosal immune activation and arthritis severity.

Several models indicate that both the presence and composition of the gut microbiota can influence arthritis development. In germ-free or antibiotic-treated mice, arthritis severity is often reduced, although this depends on the model used, the timing of microbial depletion and the extent to which the microbiota is removed (147–150). This suggests that the microbiota is not simply a background finding, but can influence the inflammatory threshold of the host. At the same time, partial depletion may have different effects from complete depletion, suggesting that the composition of the microbial community may be more important than the presence of bacteria alone (147–149, 151).

In the K/BxN spontaneous arthritis model, absence of the microbiota reduces arthritis severity, with lower numbers of Th17 cells and reduced autoantibody production. Recolonization with segmented filamentous bacteria restores intestinal Th17 responses and promotes arthritis through enhanced T-B cell interactions (151). This model is useful because it links gut colonization, Th17 induction and systemic autoantibody-driven arthritis. It should however be distinguished from the passive K/BxN serum-transfer model, which mainly reflects the effector phase of joint inflammation rather than the initiation of adaptive autoimmunity.

A similar conclusion has been drawn from the IL-1 receptor antagonist-deficient model. Mice lacking IL-1Ra develop spontaneous T-cell-mediated arthritis, but microbial colonization is required for full disease expression (150, 152). In this model, gut dysbiosis promotes expansion of Th17 cells in the lamina propria and aggravates joint inflammation. Depletion of selected taxa, including Helicobacter, attenuates arthritis severity, whereas colonization with *Bifidobacterium bifidum* has been reported to accelerate disease onset (150, 152). These data suggest that bacteria regarded as beneficial in one setting may not necessarily be protective in another.

The SKG model has also been informative for studying microbiota-driven arthritis in a genetically susceptible host. Under germ-free conditions, SKG mice show reduced arthritis development. Recolonization studies indicate that *Prevotella*-rich microbiota from RA patients, and in some settings *S. copri*, can promote intestinal Th17 responses and aggravate arthritis (153, 154). These findings are consistent with the human association between *Prevotellaceae* and early RA. However, they do not demonstrate that the same mechanism operates in all patients.

#### 3.6.2 Human-to-mouse transfer and barrier/Th17 mechanisms

Fecal microbiota transplantation (FMT) experiments provide a stronger test of causality than observational human studies. Microbiota from anti-CCP-positive individuals transferred into antibiotic-pretreated mice increased intestinal permeability, altered tight junction protein expression, expanded Th17 cells in mesenteric lymph nodes and Peyer’s patches, and aggravated CIA (147). This provides one of the more direct experimental links between pre-RA microbial communities, barrier dysfunction, mucosal Th17 skewing, and arthritis severity.

Not all transfer studies have shown the same effect. Belvončíková et al. used FMT in CIA mice with microbiota from healthy donors, new-onset RA patients, or relapsing RA patients and did not observe a significant difference in average clinical arthritis severity between donor groups (155). Some taxa, including *Paraprevotella, Allobaculum*, and *Phascolarctobacterium*, correlated positively with disease severity, whereas *Odoribacter* showed an inverse correlation. However, the study was limited by small group sizes, use of a single 16S rRNA region, and absence of sham-FMT controls (155). This shows that transfer experiments are also influenced by study design, donor selection and recipient conditioning.

The CIA model also supports the idea that microbial changes may precede clinical joint inflammation. In CIA mice, dysbiosis has been described in the preclinical phase, with changes in *Bacteroidota, Bacillota* and *Pseudomonadota*, and with altered abundance of several bacterial families (149, 150, 156, 157). These changes have been associated with Th17-related inflammatory responses. The direction and composition of dysbiosis, however, are not identical between studies. Differences in mouse strain, housing conditions, diet, induction protocol and sequencing methods are likely to account for at least part of this variation.

Specific microbial metabolites can also modify experimental arthritis*. F. prausnitzii* and *B. fragilis* are human-derived commensals, but in these studies they were administered to mice as single, defined strains rather than as part of a human fecal community. Oral administration of viable *F. prausnitzii* reduced clinical arthritis scores, histological joint damage and IL-17-producing cells in CIA mice (17). The intervention also altered SCFA profiles, with increased butyrate and changes in microbial composition*. B. fragilis* has been shown to protect against CIA, with propionate identified as an important mediator (124). These studies suggest that selected commensals can have anti-inflammatory effects in arthritis models. They do not show that the same organisms would be effective or safe as preventive treatment in humans.

#### 3.6.3 Defined taxa, strain-level effects and protective commensals

Defined colonization studies have shown that the effect of a bacterial taxon may depend on properties present at strain level. *S. copri* is the clearest example. In gnotobiotic models, mice colonized with RA-associated *S. copri* strains developed more severe arthritis and stronger Th17 responses than mice colonized with control-associated strains (20, 94). Nii et al. identified a conjugative transposon region, CTnPc, which was enriched in RA-associated *S. copri* strains and absent from control-associated strains (20). This strain-dependent effect tracks with the CTnPc genomic region described earlier (Section 3.1), reinforcing that pathogenic potential cannot be inferred from genus- or species-level identification alone.

Other members of the *Prevotellaceae* family have also been linked with arthritis development. Maeda et al. showed that colonization with *Palleniella* intestinalis induced arthritis in genetically resistant C57BL/6 mice under the experimental conditions used in that study (158). Colonized mice showed increased intestinal permeability and activation of colonic CD11b^+^CD11c^+^ myeloid cells, followed by IL-6 dependent Th17 differentiation and joint inflammation. Outer membrane vesicles from *P. intestinalis* and *S. copri* also promoted Th17 differentiation through dendritic cell activation *in vitro* (158). This provides a defined microbe-immune pathway in which barrier disruption, myeloid cell activation, and Th17-mediated inflammation are connected. Whether the same pathway is operating in human RA remains uncertain.

*Subdoligranulum* illustrates the same problem from a different angle. *S. didolesgii*, isolated from individuals at risk of RA, activated CD4^+^ T cells from RA patients and induced arthritis-like changes after transfer to germ-free mice, including joint swelling, autoantibody production and complement deposition (91). Colonization was associated with expansion of Th17 cells and formation of intestinal lymphoid follicles. By contrast, *S. variabile* has been reported to reduce arthritis severity through induction of the anti-inflammatory mediator TSG-6 (92). These observations suggest that bacteria within the same genus may have opposing effects. A *Subdoligranulum* signal in microbiome studies is therefore difficult to interpret without strain-resolved and functional data.

Some bacteria enriched in RA may also not be harmful in all settings. *Peptoniphilus gorbachii*, reported to be increased in some RA patients, improved intestinal barrier integrity and reduced arthritis severity in CIA mice (159). *P. gorbachii* administration increased expression of ZO-1 and occludin, reduced serum zonulin-family peptides, and reduced pro-inflammatory cytokine expression. The mechanisms were not fully defined, and the study used serum microbial arrays rather than comprehensive metagenomic profiling (159). Even so, the findings show that association with RA does not, by itself, demonstrate a pathogenic effect.

Probiotic-type interventions have produced mixed but useful results in animal models. Lactobacillus fermentum PC1 reduced paw swelling, synovial inflammatory infiltration, and cartilage damage in CIA mice, with reduced serum IL-12 and increased IL-4 and IL-10 (160). The timing of administration appeared to affect the size of the response. This suggests that some microbial interventions may act through immune deviation or through stimulation of regulatory pathways. It also indicates that timing, strain selection and disease stage are likely to matter.

Early microbial exposure may also modify later susceptibility to arthritis. In pristane-induced arthritis, early-life rearing experiments showed that microbiota acquired in the neonatal period altered genetic susceptibility to chronic arthritis (161). Susceptible mice reared by resistant strains developed less severe disease. This suggests that host genotype and early-life microbiota interact in shaping later inflammatory responses. At the same time, makes direct extrapolation to adult human RA difficult.

The experimental literature therefore supports the view that the gut microbiota can modify arthritis development and severity in susceptible hosts. Barrier dysfunction, Th17-skewed immunity, microbial metabolites and strain-specific bacterial factors have all been shown to influence disease expression. The strongest evidence comes from defined bacteria, gnotobiotic experiments and transfer studies. The link is less direct when animal data are used to explain heterogeneous findings in human microbiome studies.

There are important limitations to these studies. Mouse models differ in genetic background, immune trigger, microbiota composition, housing, and diet. Some models are strongly dependent on Th17 biology, while human RA involves a broader cytokine network, including TNF-α, IL-6 and IL-1β. Laboratory mouse microbiota also differs substantially from human microbiota. Experimental studies should therefore be seen as tests of biological possibility, not as direct models of human RA pathogenesis.

These limitations do not reduce the value of the models. They indicate what still needs to be tested. Future studies should use strain-resolved microbiology, defined microbial consortia, metabolite measurements and parallel assessment of barrier function, mucosal immunity, and synovial pathology. Human-to-mouse transfer experiments should include careful donor phenotyping, standardized recipient conditioning, and appropriate sham-FMT controls. Such designs would make it easier to distinguish microbial signals that may be relevant to human RA from effects that are specific to the experimental model.

## 4 Intestinal permeability in RA

### 4.1 Evidence for barrier dysfunction in human RA

While barrier dysfunction is biologically plausible in RA, pooled human data are mixed. A 2021 systematic review concluded that increased intestinal permeability is consistently observed in spondyloarthritis, whereas findings in RA are more controversial, with several studies reporting no difference vs controls (162). This heterogeneity is independent of NSAID use and assay choice (162).

In preclinical arthritis models, intestinal barrier dysfunction precedes joint disease. In models such as K/BxN, CIA, and related models, disruptions in TJs, increased zonulin-family signaling, microbial translocation, and mucosal immune activation preceded the appearance of clinical arthritis symptoms and experimental barrier stabilization decreased disease severity (68, 83, 98, 150, 158, 163, 164).

In parallel, human data indicate barrier involvement at-risk subjects and around onset. A recent study revealed that ACPA-positive, arthritis-free “pre-RA” individuals have greater circulating ZFPs when compared to HCs (165). Early RA cohorts show elevations, variably of barrier markers (ZFPs, I-FABP) and microbial-product exposure markers (LPS, LBP/sCD14), and a study using paired tissue/sera demonstrated increased intestinal permeability and markers consistent with microbial-product translocation which normalized with inflammation control (166, 167). Similarly, a 24-week prospective study reported changes in serum TJ proteins after anti-TNF therapy alongside clinical improvement (168). Thus, while common exposures such as NSAIDs and PPIs can aggravate gut barrier injury, effective disease-modifying therapies may help restore barrier integrity (166, 168, 169). Together, these human observations and preclinical experiments support a mechanistic focus on epithelial TJs, zonulin signaling, and microbial drivers of barrier injury.

Converging, but largely associative, human evidence suggests that intestinal dysbiosis and barrier dysfunction may participate in RA pathogenesis. Putative gut-to-joint connectors include microbial-product translocation and microbial nucleic-acid signatures and mucosal citrullination; however, causal proof remains limited and relies mainly on indirect biomarkers and preclinical models. Recent publications discuss this possibility: Romero-Figueroa et al. in their narrative review (170), outline multiple plausible routes, including epithelial-barrier defects, while a mechanistic review by Blenkinsopp et al. (171) highlights microbial-product translocation and mucosal citrullination as hypotheses consistent with current data.

Together, these findings reinforce a gut–joint axis in RA: a perturbed microbiome and intestinal barrier dysfunction may permit microbial-product translocation and systemic exposure to microbial molecules and local intestinal priming at disease onset (107, 123).

In human cohorts, most commercial “zonulin” ELISAs detect a mixture of zonulin-family peptides rather than pre-haptoglobin-2, and kits vary in specificity; cross-study comparisons should therefore be interpreted cautiously. We adopt the ZFP terminology when referring to serum assays and highlight that analytical choice may partly explain discrepant human findings (108, 109, 165).

Circulating ZFPs are increased in individuals at risk for RA (ACPA-positive, arthritis-free) and have been reported in pre-RA/RA, consistent with early barrier involvement (68, 165). These data support a plausible sequence whereby dysbiosis-induced zonulin release triggers TJ dysfunction and microbial-product translocation, potentially initiating arthritis in susceptible hosts (68, 165). However, other at-risk cohorts have not shown significant changes in certain barrier markers (e.g. LBP, I-FABP) before RA onset, indicating heterogeneity in preclinical barrier dysfunction (172).

Prospective pre-RA data also point to a “smoldering” gut state: in FDRs, microbiome perturbations and subtle mucosal inflammation (e.g., fecal calprotectin) were enriched among those who later developed RA, reinforcing a mucosal origin without directly demonstrating permeability (24).

Multiple circulating or fecal biomarkers indicate barrier dysfunction and/or systemic exposure to microbial products in RA. In cross-sectional cohorts, ZFPs and I-FABP are frequently elevated in active disease; LPS is often increased and associates with inflammatory activity, whereas LBP and sCD14 show mixed results across studies. Some of these markers are already elevated in at-risk patients or near onset with the most consistent activity links reported for ZFPs, LPS and I-FABP (68, 167, 173). Conversely, in one treatment-naïve early-RA cohort, sCD14 and LBP were not elevated when compared with controls. This occurred despite evidence of microbial-product exposure or microbial nucleic-acid signatures, underscoring heterogeneity in the timing, compartmentalization, and magnitude of barrier-related abnormalities (107). Consistently, a 2025 study (174) reported that fecal ZFPs (reported as ‘fecal zonulin’ in the original assay) were elevated in active RA and helped to distinguish high-disease-activity patients. The fecal analytes outperformed plasma readouts in the same study: fecal zonulin, FABP2 and D-lactate discriminated active from remission patients (AUCs ~0.90), whereas plasma counterparts performed poorly. Fecal zonulin also separated difficult-to-treat from naïve cases, while plasma HIF-2α, not plasma zonulin, drove classification. This compartmental split introduces a practical “biomarker dissociation” concept, i.e., circulating levels may not faithfully reflect gut-local barrier status, arguing for fecal markers when interrogating mucosal integrity in RA (174).

### 4.2 From barrier damage to immune activation

When the intestinal barrier is compromised, microbial products and molecular signatures, rather than viable organisms, may be detected beyond the gut. This is mechanistically distinct from septic arthritis: in RA the relevant process is an immune response to microbial antigens (bacterial DNA, peptidoglycan, citrullinated proteins), not colonization of the joint by live bacteria. Consistent with this, newly diagnosed, treatment-naive RA cohorts show elevated plasma bacterial 16S rRNA gene copies that track with the dysbiotic fecal profile, and bacterial 16S rRNA fragments have been detected in synovial fluid and tissue under stringent low-biomass conditions (175), Such findings indicate that microbial nucleic acids, not necessarily viable bacteria, can reach the joint; demonstrating true translocation of live organisms would require rigorous contamination controls and viability assays. Microbial products detected systemically, such as LPS and peptidoglycan, can engage pattern-recognition receptors(PRRs) such as TLR4 (with LBP and CD14 as cofactors) on monocytes/macrophages, inducing TNF-α, IL-1, IL-6, and cytokines which drive RA pathology. Human cohorts often show elevated LPS/LBP/sCD14 in association with disease activity, though results vary by cohort; LBP correlates with activity in several studies (173).

Dendritic cells and macrophages carrying gut microbial antigens can traffic to lymphoid organs (and potentially joints), present antigens to T cells, and help break tolerance. Gut microbes can also drive mucosal Th17 responses that disseminate. In the K/BxN model, SFB adhering to ileal epithelium induce a robust Th17 response; SFB monocolonization of GF K/BxN mice is sufficient to trigger arthritis, whereas GF mice remain non-arthritic, highlighting host–microbe synergy (176–178).

Rheumatoid arthritis involves dysregulated adaptive and innate immune responses, including alterations in Th17/Treg pathways, but these occur within a broader inflammatory network dominated by TNF-α, IL-6, IL-1β, and other cytokine-driven mechanisms. Gut-derived signals are known to skew the Th17/Treg balance toward inflammation (preclinical evidence: SFB and other dysbiotic taxa promoting Th17). In humans, dysbiosis is associated with Th17-prone signatures, and loss of microbial metabolites that support Tregs may compound this. Treatment-naive RA patients show lower levels of fecal tryptophan-derived AhR ligands (e.g., kynurenic and xanthurenic acids, 3-HAA, 5-HIAA), likely limiting mucosal Treg induction and tolerance (23, 28, 177).

The observed depletion of fecal tryptophan-derived AhR ligands in treatment-naive RA provides a plausible link between dysbiosis and a shifted mucosal regulatory tone via AhR signaling (23). Wu et al. (179) identified dysregulated tryptophan metabolism in both RA and Pre-RA individuals and FMT mice, marked by a shift toward the kynurenine pathway and reduced activity in serotonin and indole pathways. In a recent review, Kong et al. (128) suggested that beyond the well-known role of tryptophan metabolites, histamine might act as a central mediator linking gut microbiota-derived metabolites with immune dysregulation and joint pathology in RA. Histamine’s interaction with H1R, H2R, and H4R triggers the activation of synovial fibroblasts, cytokine production, and osteoclastogenesis, thereby worsening synovitis and bone erosion in RA. This axis is both bidirectional and influenced by neuroimmunomodulation (128).

In summary, increased intestinal permeability may increase exposure to luminal antigens and microbial products, (rather than necessarily viable whole bacteria), triggering chronic immune activation and potentially breaking immune tolerance to self. An excellent review by Kuhn et al. (180) pointed out that “intestinal permeability” and “bacterial translocation” are distinct processes with different consequences for the immune system, and urged caution in using the term “leaky gut” as a general term. Here, increased intestinal permeability refers to epithelial barrier dysfunction, microbial-product translocation refers to LPS/peptidoglycan entry, and bacterial dissemination is reserved for viable bacteria or rigorously controlled intact bacterial nucleic-acid detection. Accordingly, the detection of permeability markers or microbial products should not be interpreted as evidence of viable bacterial dissemination to the joint. This distinction is important for interpreting biomarker data and for designing interventional studies (180).

Zonulin-related biomarker work also need tighter analytical definition. Commercial assays sold as “zonulin” do not necessarily measure the same target. Immunoprecipitation-mass spectrometry studies have shown that several widely used kits do not quantify pre-haptoglobin-2 specifically, but may detect other zonulin-family or structurally related proteins (108). Future studies should therefore state the analyte explicitly (108, 181). If the intention is to measure pre-haptoglobin-2, targeted mass spectrometry or standardized monoclonal antibody-based pre-HP2 ELISAs should be preferred (108, 182). If a broader family signal is measured, the result should be reported as a zonulin-family peptide readout rather than as zonulin without qualification (108). Future studies should therefore state the analyte explicitly (108, 181). Where feasible, these assays should also be benchmarked against a functional permeability test such as lactulose/mannitol and not treated as self-validating surrogates (181). In human RA cohorts, the most informative design would be paired fecal and serum or plasma sampling at the same visit, ideally before treatment changes. Fecal samples should be used to assess local permeability or mucosal injury markers, together with fecal calprotectin as a companion marker of intestinal inflammation (181). Serum or plasma should be used to measure circulating markers of epithelial injury, microbial-product exposure, and systemic inflammation.This design would make it easier to distinguish local intestinal events from secondary systemic signals and would improve comparison across cohorts (181).

Rheumatoid arthritis joints contain immune cells (including T cells) and autoantibodies that appear to recognize microbial antigens first encountered at mucosal surfaces, supporting a mucosal origin of RA (9, 28). However, this process is not unidirectional. Although increased intestinal permeability is often observed before or around RA onset, established inflammation can in turn exacerbate barrier dysfunction: RA-relevant cytokines (e.g., TNF-α, IFN-γ, IL-17) impair TJs and can promote epithelial injury. Experimental data further show that epithelial stress pathways (e.g., necroptosis) increase permeability, while barrier-protective signaling reduces arthritis severity. Together, these observations outline a vicious cycle in which dysbiosis compromises the intestinal barrier, permitting microbial-product translocation that activates immunity, driving systemic inflammation that in turn further damages the barrier. This bidirectional model supports the notion of gut-directed interventions (barrier-stabilizing and microbiota-modulating) as potential adjuncts in RA management.

Even in the absence of gastrointestinal symptoms, some patients with RA may show evidence of intestinal immune activation. In early DMARD-naïve RA, ileal or colonic biopsies have revealed subclinical mucosal immune changes, including infiltrates of T cells, B cells, and macrophages. Although older biopsy studies reported focal gut inflammation in patients classified as RA (183, 184), these findings should be interpreted cautiously because historical diagnostic categories may not fully correspond to current RA and SpA classification frameworks. More recent analyses support the concept that the gut may represent an immunologically active site in at least a subset of RA patients (185). These clinical findings and experimental mechanisms align with a bidirectional gut–joint axis, where inflammation both follows and further compromises epithelial barrier integrity. Interestingly, Moran et al. (186) demonstrated that a single injection of adjuvant into the joint triggers a coordinated cell and molecular response in intestinal tissues. In the ileum, there was a rapid secretion of mucus and a silencing of T-cell pathways, while in the colon, there was a transient upregulation of macrophages and a broader suppression of metabolic transcripts (186).

Rheumatoid arthritis-relevant inflammatory mediators can directly undermine the intestinal barrier. In experimental systems, exposure of intestinal epithelial cells to cytokines such as TNF-α or IFN-γ disrupts TJs and reduces occludin and ZO-1 expression. This is analogous to IBD, where TNF-α activates myosin light-chain kinase to loosen junctions. *In vivo*, arthritis-associated inflammation induces epithelial necroptosis (↑RIPK3/MLKL) and increases gut permeability at disease onset. Moreover, activation of epithelial hypoxia-inducible factor-1α (HIF-1α) blocks RIPK3-mediated necroptosis, preserves barrier integrity, and attenuates arthritis severity (158).

While we present dysbiosis and epithelial-intrinsic pathways (glycosylation, Hippo–YAP/TAZ) as upstream drivers of barrier injury, the reverse direction is also possible and supported by data (187, 188). Taken together, these data support bidirectionality: barrier injury can amplify systemic inflammation, and systemic inflammatory tone can further destabilize epithelial homeostasis (187, 188). In parallel, YAP/TAZ is robustly active in RA synovium, and inflammatory tone interfaces with Hippo signaling, suggesting that systemic inflammation can reprogram gut epithelial YAP/TAZ activity and glycosylation to impair junctional maturation (70). Thus, barrier dysfunction is both a potential trigger and a likely consequence of systemic autoimmunity, a self-amplifying cycle rather than a linear cascade. In parallel, epithelial IL-10R signaling restrains barrier dysfunction (163).

Anti-inflammatory signaling helps to maintain barrier integrity during arthritis. In an experimental study, mice lacking IL-10R specifically on intestinal epithelial cells developed a hyper-permeable gut and more severe arthritis, indicating that epithelial IL-10R signaling restrains inflammation and reinforces the barrier (163). Furthermore, IFN-γ antagonized this protective loop: exposure to IFN-γ downregulated epithelial IL-10R, undermining IL-10–mediated barrier protection. This interplay suggests that, in RA’s inflammatory milieu, diminished IL-10 signaling and excess IFN-γ can jointly compromise epithelial integrity (163). Overall, immune responses characteristic of RA such as excess TNF-α, IL-17, and IFN-γ alongside insufficient IL-10, can directly disrupt intestinal TJ structure and function, amplifying the initial microbiota-driven barrier injury (68, 158, 163, 188).

The gut–joint axis operates as a self-reinforcing mechanism because a compromised gut barrier initiates autoimmune inflammation, which then intensifies to damage the barrier further.

## 5 Discussion

### 5.1 Strength of the current evidence

Available evidence underscores the significance of the gut-joint axis in RA; however, the strength of the conclusions depends on the type of evidence. Preclinical models provide strong mechanistic support and demonstrate that dysbiotic bacterial communities, gut barrier disruption, and specific bacterial strains or their metabolites can modify the severity of arthritis and modulate mucosal immunity (68, 83, 124, 147, 189). However, although numerous human studies have suggested associations between RA and changes in the gut microbiota composition, altered microbiome metabolite profiles, and biomarkers of gut barrier dysfunction, no single, reproducible microbial signature or disease-defining intestinal permeability abnormality has been identified across populations (18, 23, 24, 28, 190). In summary, the current literature appears to support the notion that the gut plays a contributing and context-dependent role in the pathogenesis of RA; however, there is no universal, defining intestinal abnormality in this disease.

The main conclusion from human studies is that maintaining a static model of preclinical dysbiosis over many years is increasingly untenable. The evidence reviewed here is more consistent with a dynamic and stage-dependent gut–joint model than with a single, stable microbial signature of RA. In this model, microbial instability may emerge in a late peri-onset window in selected at-risk individuals, but this interpretation remains preliminary and requires validation in larger longitudinal cohorts (25). This model reconciles previous conflicting reports, especially those concerning taxa associated with *Prevotella* spp. Cross-sectional studies in at-risk populations, such as SCREEN-RA, have not consistently shown between-group differences in microbiome diversity or taxonomic composition across preclinical stages (24). However, longitudinal data on individuals positive for anti-CCP antibodies suggest that microbiome instability may not manifest until the late preclinical period, approximately ten months prior to arthritis onset (25). The contrast between the peri-onset signal described by Rooney et al. and the lack of significant differences in the SCREEN-RA study argues against a model of stable dysbiosis driven by a single pathobiont. Instead, the data indicate a narrow ecological window where disruption of the gut barrier and activation of mucosal immunity may have the greatest pathogenetic significance (24, 25).

### 5.2 Methodological limitations and reasons for inconsistency

This framework helps explain the heterogeneity of the human literature. Gut microbiome studies in RA are difficult to compare because they differ in geography, diet, smoking status, drug exposure, disease stage, and analytical platforms (23, 81, 97). Treatment appears to be particularly important in this regard. Many DMARDs and other commonly used medications can modify the gut microbiome, whereas NSAIDs and PPIs can affect gut barrier integrity and the relationship between the microbiome and intestinal permeability (81, 97, 98).

Another challenge is methodological issues that further complicate comparisons between individual studies. Many studies are cross-sectional, sample sizes are small, 16S-based taxonomic resolution is limited, and commercially available “zonulin” assays often detect a mixture of zonulin family peptides rather than a single, validated analyte (108, 109, 165). These limitations likely contribute to the lack of a consistent α-diversity signal independent of disease stage and the variability observed in permeability and translocation markers (107, 110, 168, 191).

Despite these limitations, several converging observations from human studies deserve to be highlighted. Various studies in RA patients have observed an enrichment of taxa such as organisms related to *Prevotella*, *Collinsella*, and *Eggerthella*, along with a depletion of butyrate-producing commensals, including *Faecalibacterium prausnitzii*, although none of these shifts is universal (19, 23, 24, 83, 107). Recent data on early-stage RA patients without prior treatment are particularly informative because they integrate dysbiosis, epithelial damage, and microbial nucleic-acid signatures, shown by elevated plasma 16S rDNA levels.in the same cohort, with increased *Escherichia*, decreased butyrate producers, and increased I-FABP (107). At the same time, metabolomic studies suggest depletion of AhR ligands derived from tryptophan and other small molecules important for the regulation of mucosal immunity in these patients, supporting the biological plausibility of an intestinal influence on the Treg/Th17 balance (4, 23, 179). Nevertheless, these findings remain largely correlational in humans, and the directionality of many of the observed associations remains to be elucidated.

Research findings on intestinal permeability vividly illustrate this concept. Preclinical models of intestinal inflammation highlight the role of intestinal barrier dysfunction. Altered TJ integrity, increased zonulin-family signaling, enhanced microbial-product translocation, and mucosal immune activation may precede clinical arthritis and can be experimentally modified (68, 83, 98, 158, 163). However, human studies have produced mixed evidence. Some studies involving risk groups and early-stage RA show elevated levels of ZFP, I-FABP, LPS-related markers, or circulating bacterial nucleic acids in subjects, while others fail to detect consistent abnormalities (98, 107, 165, 167, 180). Therefore, it is important not to lump several related but distinct processes into a single concept of “leaky gut.” Increased intestinal permeability, translocation of microbial products, detection of microbial nucleic acids, and spread of viable whole bacteria are not equivalent phenomena and should not be understood interchangeably (180). In particular, claims regarding the joint seeding of whole bacteria should not be based solely on permeability markers, and results regarding low biomass require particularly rigorous controls (94, 175, 180).

One important conclusion to be drawn from the current state of knowledge is that the gut-joint axis is likely to be bidirectional. While dysbiosis and epithelial-specific mechanisms may contribute to early barrier damage, inflammation that develops during RA may further destabilize epithelial homeostasis. Tight junctions may be directly impaired by TNF-α, IFN-γ, and IL-17, whereas epithelial necroptosis and impaired IL-10R signaling can exacerbate barrier dysfunction and increase intestinal permeability in experimental systems (158, 163). This suggests that the self-perpetuating cycle in which dysbiosis and barrier damage increase the host’s exposure to intestinal lumen antigens and microbial products, thereby promoting chronic immune activation, which, in turn, causes further barrier damage (158, 163, 170, 187, 188). This model aligns with the observation that barrier-related indices may improve with effective control of inflammation, even if markers of microbial-product exposure or microbial nucleic-acid signatures exhibit heterogeneous behavior over time (166, 191).

Sex should also be considered in future studies, because RA incidence differs between women and men and sex-related differences in microbiota composition, epithelial barrier regulation, and mucosal immune responses may influence gut-joint axis signals. Most available RA microbiome studies have not been sufficiently powered or stratified to assess these effects (192).

Molecular mimicry, altered tryptophan signaling, strain-specific pathogenicity of *Prevotella* bacteria, and barrier-mediated immune spillover are plausible hypotheses supported to varying degrees by data from human associative studies and preclinical experiments (20, 23, 83, 124, 143, 147, 179). The RA-specific role of dysregulation of the Hippo–YAP/TAZ pathway or defects in mucin glycosylation in the gut may prove to be significant; however, at present it remains speculative and is based mainly on models of intestinal barrier damage or other inflammatory diseases, rather than on direct studies of intestinal tissue in RA (42, 70, 71, 73).

### 5.3 Future research directions

Several conclusions emerge from this literature review. Future research should not be limited to single-time-point survey studies but should move toward serial sampling in at-risk populations, particularly in the peri-onset window, as suggested by recent longitudinal studies (24, 25). Microbiome studies should increasingly emphasize strain-level and function-level resolution rather than solely genus-level description, as organisms grouped within the same genus may have divergent immunological effects, as exemplified by taxa related to *Subdoligranulum* and *Prevotella* (20, 91, 92, 193, 194). Microbiome composition studies should be integrated with metabolomics, barrier biomarkers, and mucosal immune readouts, with careful control of exposure to factors such as medications, smoking, and diet (23, 81, 97, 98). Finally, interventional studies targeting the gastrointestinal tract in at-risk populations are needed to determine whether stabilizing the gut barrier or correcting late ecological instability can significantly alter the clinical course of RA (25, 68, 124, 147).

### 5.4 Translational outlook: biomarkers and timing of gut-directed strategies

The proposed late period of microbiome instability before the onset of arthritis may have translational relevance, although at present it should be viewed as preliminary. It gives a reason to study at-risk individuals, rather than a reason to intervene clinically. It is also unlikely that a single marker will be sufficient. A more useful approach would probably combine anti-CCP positivity and arthralgia with repeated stool microbiome profiling, including changes in *Prevotellaceae* or *S. copri* abundance, together with fecal ZFPs, FABP2, D-lactate, and fecal calprotectin (24, 25, 109, 174). Serum I-FABP, LPS-related markers, LBP, and sCD14 could also be included, but these are perhaps better interpreted as systemic readouts rather than as direct evidence of local mucosal injury (107, 108, 165, 167, 173). If the main question is gut-local barrier dysfunction, fecal markers are likely to be more informative (174).

Dietary modification would seem the most appropriate intervention to test first. A high fiber, Mediterranean-type, or more broadly plant-rich diet aimed at increasing SCFA production is feasible, acceptable, and likely to be safer than more invasive manipulation of the microbiome (12, 119–121). Prebiotics could be studied in the same setting. Probiotics are also of interest, although strain selection remains a major problem, as bacteria within the same genus may have quite different biological effects (20, 91, 92). For this reason, butyrate or other postbiotic approaches may be easier to standardize than live organisms, although there are still no human data showing that they prevent RA (17, 124). Larazotide is also of interest, given its effects on tight-junction regulation and its previous testing in celiac disease (195). Its use in pre-RA, however, would require separate safety and dose-finding work. Fecal-microbiota transplant would not seem suitable as an initial prevention strategy in this setting, given donor variability and safety concerns.

A practical study could enroll anti-CCP-positive individuals with arthralgia who also show at least two abnormal gut-related readouts on repeated sampling (24, 25). Participants could then be randomized to usual care or to a structured dietary and prebiotic program, with or without an additional butyrate-based postbiotic arm. A larazotide arm would be more appropriate only after preliminary safety data are available. Follow-up should last at least 12 months, with repeated stool and blood sampling every 2–3 months. Outcomes should include progression to clinical arthritis or classifiable RA, ultrasound or MRI evidence of synovitis, changes in autoantibody titers, fecal barrier markers, microbiome instability, microbiome instability, SCFA and tryptophan metabolites, and immune readouts (23, 25, 68, 165, 174). Such a design would test whether intervention during the suspected peri-onset period can alter disease development, rather than only shift biomarkers.
